# The Potential Role of Verapamil Against Fluconazole‐Induced Torsade de Pointes: A Critical Review

**DOI:** 10.1002/hsr2.72288

**Published:** 2026-05-13

**Authors:** Noha E. Abdel‐Razik, Hayder M. Al‐Kuraishy, Ali I. Al‐Gareeb, Maryam Magdy Soliman, Athanasios Alexiou, Marios Papadakis, Mubarak Aluwaili, Gaber El‐Saber Batiha

**Affiliations:** ^1^ Department of Medical Laboratory Technology, College of Nursing & Health Sciences Jazan University Jazan Saudi Arabia; ^2^ Department of Pharmacology, Toxicology and Medicine, College of Medicine Al‐Mustansiriyah University Baghdad Iraq; ^3^ Faculty of Pharmacy Menoufia University Shebin El‐Kom Menoufia Egypt; ^4^ University Centre for Research & Development Chandigarh University Mohali Punjab India; ^5^ European Academy of Sciences and Arts Austria; ^6^ Medical Department, Faculty of Life Sciences University of Thessaly Viopolis Larissa Greece; ^7^ Department of Internal Medicine, College of Medicine Jouf University Sakaka Saudi Arabia; ^8^ Department of Pharmacology and Therapeutics, Faculty of Veterinary Medicine Damanhour University Damanhour Al Beheira Egypt

**Keywords:** antifungal, fluconazole, Torsade de Pointes (TdP), verapamil

## Abstract

**Background and Aims:**

Fluconazole (FZL) is a broad‐spectrum antifungal drug associated with certain serious adverse effects such as polymorphic ventricular arrhythmia due to QT prolongation. Torsade de Pointes (TdP) is a unique type of polymorphic ventricular arrhythmia due to QT prolongation. It has been shown that the calcium‐channel blocker verapamil is effective in the management of TdP. Therefore, the present critical review aims to discuss and explain the possible role of verapamil in the prevention of FZL‐induced TdP.

**Methods:**

Databases of Scopus, Cochran, Embase, PubMed, and CENTRAL were examined to recognize suitable publications. Screened articles were designated rendering to exact eligibility criteria including original articles such as prospective and retrospective studies recognizing the role verapamil in the prevention of FZL‐induced TdP.

**Results:**

The use of FZL is linked with the development of TdP and prolongation of QT, and verapamil use could be effective in the management of TdP. In addition, verapamil has antifungal effects, affects the pharmacokinetics and pharmacodynamics of FZL. Verapamil inhibits the propagation of TdP by inhibiting the deregulation of repolarization early after depolarization in the heart.

**Conclusions:**

This critical review suggests that combination of verapamil with FZL leads to more beneficial effects by increasing the antifungal activity of FZL with a significant reduction the development of TdP which is a serious adverse effect of FZL.

## 1 Background

**Figure 1:**
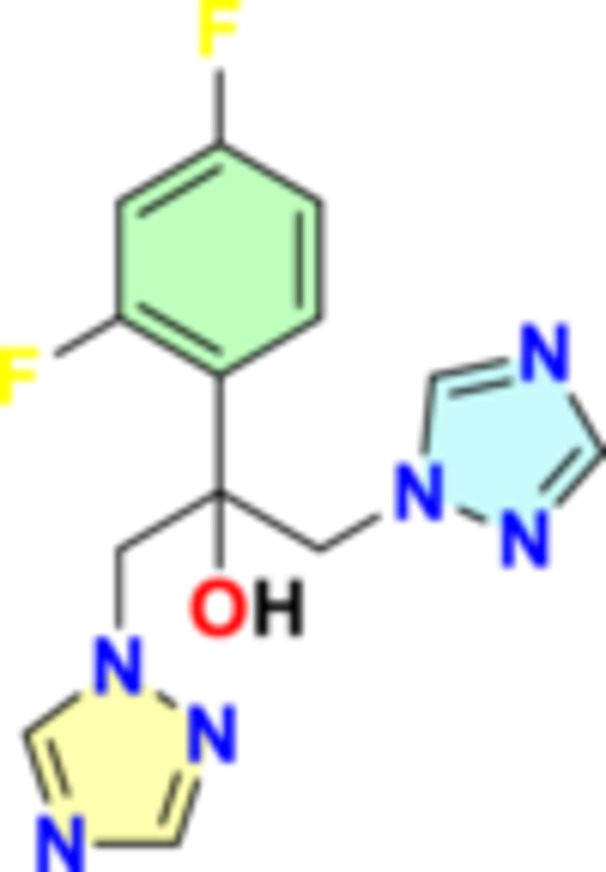
Fluconazole structure.

Fluconazole (FZL) is a broad‐spectrum antifungal drug (Figure 1), used in the treatment of different fungal infections including candidiasis, coccidiodomycosis, blastomycosis, cypococcosis, dermatophytosis, histoplasmosis, and *Pityriasis Versicolor* [1]. Of note, FZL is commonly used in immunocompromised patients with severe neutropenia and after organ transplantation to prevents opportunistic fungal infections [2]. Usually, FZL is taken by mouth or parentally according to the severity of fungal infection [2].

Notably, FZL was patented in 1981, became commercially available in 1988, and was regarded as one of the World Health Organization's Essential Medicines [3]. The most common serious adverse effects of FZL are acute liver injury, increased liver enzymes, convulsion, and QT prolongation [4]. FZL is contraindicated during pregnancy as it causes abortion and birth defects [5].

**Figure 2:**
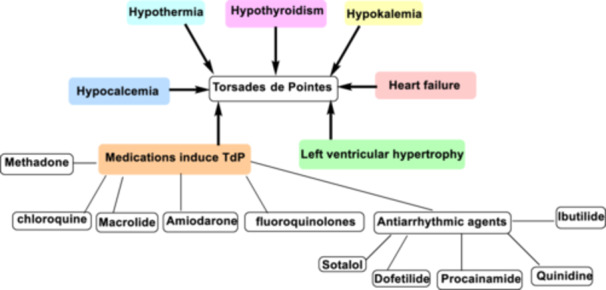
The risk factors for the development of TdP. The risk factors for the development of TdP are hypokalemia, hypothyroidism, hypothermia, hypocalcemia, heart failure, and left ventricular hypertrophy. Medications that induce the propagation of TdP are chloroquine, macrolide antibiotics, amiodarone, methadone, and fluoroquinolones. Moreover, TdP may also develop due to using some antiarrhythmic agents such as sotalol, dofetilide, procainamide, ibutilide, and quinidine.

QT prolongation represents repolarization defects after the complete heart cycle causing the development of ventricular arrhythmia. Torsade de Pointes (TdP) is a unique type of polymorphic ventricular arrhythmia due to QT prolongation [6]. TdP with specific electrocardiogram (ECG) characteristics was initially described by the French physician in 1966 [7]. Of note, acquired QT prolongation is developed in response to different diseases and medications [8, 9]. The risk factors for the development of TdP are hypokalemia, hypothyroidism, hypothermia, hypocalcemia, heart failure, and left ventricular hypertrophy [8]. Medications that induce the propagation of TdP are chloroquine, macrolide antibiotics, amiodarone, methadone, and fluoroquinolones. Moreover, TdP may also develop due to using some antiarrhythmic agents such as sotalol, dofetilide, procainamide, ibutilide, and quinidine [9] (Figure 2). However, congenital long QT syndrome (LQTS), which is a disorder of myocardial repolarization defined by a prolonged QT interval on ECG, can cause ventricular arrhythmias and sudden cardiac death. LQTS is divided into 17 subtypes, with LQT1, LQT2, and LQT3 being the most common forms. Congenital LQTS is diagnosed by a series of 12‐lead ECG recordings, and calculation of the LQTS diagnostic score known as the Schwartz score [10, 11].

The development of TdP leads to severe palpitation, headache, dizziness, fainting, and sudden cardiac death, however, most of TdP episodes are reverted to normal rhythm [7].

Commonly, TdP is managed urgently by electrical cardioversion, infusion of magnesium sulfate, mexiletine, and/or β blockers with correction of electrolyte disturbances [12]. It has been shown that verapamil could be used in the management of TdP. In addition, verapamil affects the pharmacokinetics and pharmacodynamics of FZL [13, 14].

Hence, the existing critical review aims to discuss and explain the conceivable role of verapamil in the prevention of FZL‐induced TdP.

## 2 Methods and Search Strategy

In the present review, databases of Scopus, Cochran, Embase, PubMed, and CENTRAL were scrutinized by two independent reviewers to observe the applicable publications. Screened articles were nominated interpreting to exact eligibility criteria including original articles such as prospective and retrospective studies distinguishing the causal relationship between FZL and TdP, distinct from other diseases, using animal and human studies. The descriptors using the MeSH database are as follows (Fluconazole AND Torsade de Pointes), (verapamil AND Torsade de Pointes). All articles according to the inclusion criteria were evaluated. Nonetheless, reviews, letters, articles other than English language were excluded.

### 2.1 Fluconazole and Risk of TdP

**Figure 3:**
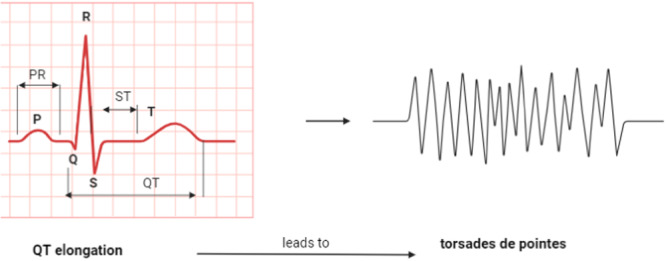
QT elongation leads to TdP.

Different studies revealed that treatment with FZL may increase the risk for the development of TdP. In a reported case study, a 33‐year‐old woman with a history of systemic lupus erythematosus treated with FZL because of *Candida albicans* pneumonia developed TdP within 1 week of treatment [15]. The risk of TdP was not correlated with the prolongation of QT, suggesting that FZL could be the possible cause for the development of TdP. Besides, FZL even at low doses may increase the risk of QT prolongation and development of TdP [16] as we can notice in Figure 3. Therefore, serial ECG measurements are recommended during treatment with FZL. Gandhi and colleagues observed that a combination of FZL and levofloxacin augments the risk of TdP and prolongation of QT [17]. Thus, the combination of FZL with other medications that prolong QT interval should be avoided to prevent the development of TdP. These observations suggest that use of FZL is linked with the development of TdP and prolongation of QT.

Conversely, a prospective study involving 150 patients with cryptococcal meningitis treated with FZL 1200 mg/day illustrated that baseline QT interval did not differ following seven treatments with FZL [18]. This controversial study proposes that FZL does not cause significant QT prolongation and development of TdP. Nevertheless, it is imperative to accentuate, as with all drugs with the probable to prolong QT interval, that it is still important to screen and accurate any electrolyte imbalance and to sidestep, anywhere promising, associated drugs that may also cause QTc prolongation.

The underlying mechanism of QT prolongation and the development of TdP is due to the dysfunction of ventricular ion channels which are involved in the process of repolarization [19]. Clinically, electrolyte disturbances, advanced age, female sex, and the underlying cardio‐metabolic disorders increase the risk of QT prolongation and TdP [20]. FZL induces QT prolongation by affecting specific ion channels in the heart. FZL can change normal ECG, lead to QT prolongation and development of TdP [19, 20].

Furthermore, genetic predisposition may increase the risk of FZL‐induced QT prolongation and propagation of TdP [18]. Also, FZL inhibits the ether‐a‐go‐go‐related gene (hERG) K^+^‐channel in a concentration‐dependent manner causing QT prolongation [21]. Dysfunction of cardiac hERG may lead to induce the development of QT prolongation, thus hERG activators and inhibitors may affect cardiac action potential and effective refractory period. However, suppression of hERG K^+^‐channel may cause ventricular arrhythmia and sudden cardiac death. Also, hERG K^+^‐channel activators modulate cardiac action potential and could be effective antiarrhythmic agents against the development of QT prolongation and propagation of TdP [22, 23]. Ledford and colleagues illustrated that disruption of cardiac hERG K^+^‐channel results in intense progression of long‐QT syndrome [24]. Therefore, FZL through inhibition of cardiac hERG K^+^‐channel may induce QT prolongation and propagation of TdP. Moreover, FZL may also affect other ion channels such as sodium and calcium channels, further interfering with cardiac electrical activity and increasing the risk of QT interval prolongation [22, 23]. Consistently, prolonged exposure of fetus to FZL during pregnancy induces permanent alterations of cardiac electrophysiology including QT prolongation which might due to distortion of cardiac ion channels [25].

### 2.2 Role of Verapamil and Flucanazole‐Induced TdP

**Figure 4:**
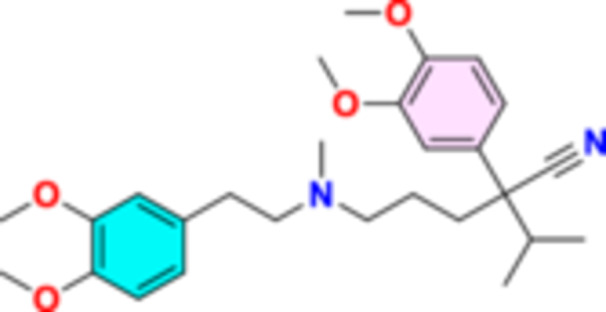
Verapamil structural formulae.

**Figure 5:**
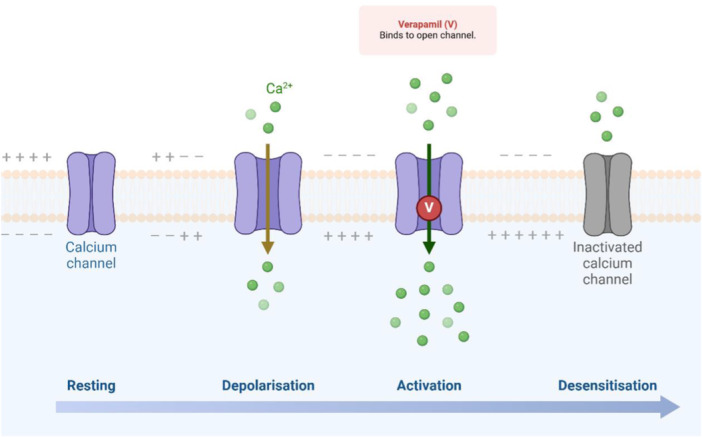
Effect of verapamil on voltage‐gated Ca^2+^‐channel. Verapamil acts by blocking of active voltage‐gated Ca^2+^‐channel with no effect on these channels during the resting state. The effect of verapamil is reduced during the desensitization phase of Ca2^+^‐channel because of limited Ca^2+^ transport.

Verapamil is a calcium‐channel blocker (Figure 4) that commonly used in the treatment of angina pectoris, hypertension, and supraventricular tachycardia as well as the prevention of cluster headaches and migraine [26, 27]. The most common adverse effects of verapamil are constipation, muscle pains, and allergic reactions [28]. Verapamil was approved in the USA in 1981 and is considered one of the World Health Organization's Essential Medicines [29]. Verapamil acts by blocking of active voltage‐gated Ca^2+^‐channel with no effect on these channels during the resting state (Figure 5). Voltage‐gated Ca^2+^‐channel is highly expressed in atrioventricular and sinoatrial nodes, thus it is used as a class IV antiarrhythmic agent [30]. As well, verapamil exerts antiarrhythmic through inhibition of voltage‐gated K‐channel [31]. Notably, verapamil inhibits the release of calcitonine gene‐related peptide (CGRP) thus; it is effective in the prophylaxis of migraine [26]. Verapamil is characterized by the high volume of distribution despite higher plasma protein binding, metabolized by the liver to active metabolites [32].

**Figure 6:**
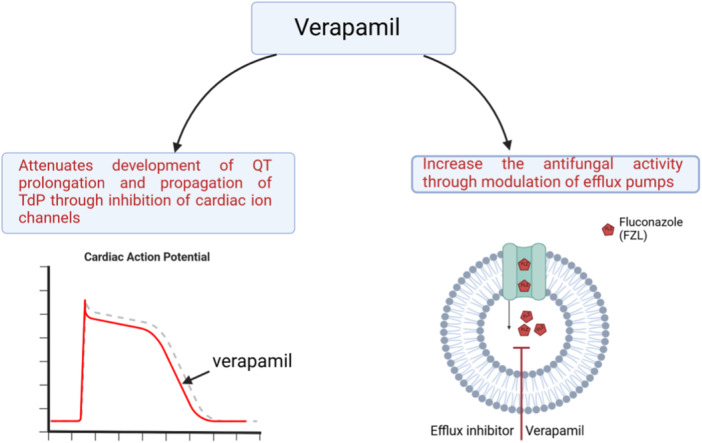
Beneficial effects of verapamil. When verapamil is used in combination with fluconazole, it increases the antifungal effect and interferes with QT prolongation and the development of TdP.

On the other hand, verapamil may increase the effect of FZL against *C. albicans* through inhibition of efflux pump in synergistic effects [27]. Importantly, verapamil attenuates FZL resistance by *Candida glabrata* [33]. Yu and colleagues illustrated that verapamil alone or in combination with FZL was effective against resistant *C. albicans* [34]. Moreover, verapamil exerts antifungal effects against non‐*C. albicans* species [35]. These observations suggest that the combination of verapamil with FZL leads to more beneficial effects by increasing the antifungal activity of FZL (Figure 6).

Despite of this beneficial effect of this combination, Alhasson and colleagues found that this combination may lead to symptomatic bradycardia needs intensive care admission [36]. Of note, verapamil inhibits the propagation of TdP through inhibition deregulation of repolarization and early depolarization in the isolated heart [37]. Also, verapamil attenuates the development of TdP in patients with heart block [38]. Various reported case studies showed that verapamil use was associated with noteworthy suppression of the development of TdP in patients with heart block and LQTS [39, 40]. Guillen and colleagues demonstrated that induced TdP is effectively treated by verapamil [41].

These findings proposed that a combination of verapamil with FZL leads to more beneficial effects by increasing the antifungal activity of FZL with significant reduction in the development of TdP which is a serious adverse effect of FZL as shown in (Figure 6).

The present critical review had several limitations including the limited clinical studies, and the serial measurements of ECG changes of FZL alone and in combination with verapamil were not evaluated. As well, biomarkers of arrhythmia‐induced cardiac injury were not evaluated.

## 3 Methods

A comprehensive literature search was conducted to identify relevant studies evaluating the role of verapamil in the prevention and management of FZL‐induced TdP. The databases PubMed, Scopus, Embase, Cochrane Library, and CENTRAL were searched from inception until [May 2025]. The search strategy included combinations of the following keywords: fluconazole, verapamil, torsade de pointes, QT prolongation, and long QT syndrome.

Eligible studies included original research articles, including prospective and retrospective studies, experimental studies, and relevant clinical reports that investigated the relationship between FZL, QT prolongation, TdP, and the potential protective or therapeutic role of verapamil.

Articles not published in English, conference abstracts without full text, editorials, and studies not directly related to the study objective were excluded. Relevant articles were screened based on title and abstract, followed by full‐text review.

## 4 Conclusions

Verapamil may increase the antifungal activity of FZL through the modulation of efflux pumps. Besides, verapamil attenuates the development of QT prolongation and propagation of TdP through inhibition of cardiac ion channels. Taken together, verapamil may be effective in increasing FZL activity with attenuating FZL‐induced TdP. However, the exact molecular mechanism of verapamil against QT prolongation and TdP development was not estimated. Therefore, additional studies are warranted in this concern.

## Author Contributions

Hayder M. Al‐Kuraishy, Ali I. Al‐Gareeb, Mubarak Aluwaili, and Gaber El‐Saber Batiha conceptualized the manuscript. wrote, edited, and reviewed the main text and approved the final edition of the manuscript. Noha E. Abdel‐Razik, Maryam Magdy Soliman Gaber El‐Saber Batiha, Athanasios Alexiou and Marios Papadakis prepared the figures, wrote, corrected, amended, and approved the final edition of the manuscript.

## Disclosure

Dr. Maryam Magdy Soliman affirms that this manuscript is an honest, accurate, and transparent account of the study being reported; that no important aspects of the study have been omitted; and that any discrepancies from the study as planned (and, if relevant, registered) have been explained. The authors declare that the research was conducted in the absence of any commercial or financial relationships that could be construed as a potential conflict of interest.

## Ethics Statement

This article does not contain any studies with human participants or animals performed by any of the authors.

## Consent

The authors have nothing to report.

## Conflicts of Interest

The authors declare no conflicts of interest.

## Data Availability

The data that support the findings of this study are available on request from the corresponding author. The data are not publicly available due to privacy or ethical restrictions.
